# Antibacterial effect of 3-*p-trans*-coumaroyl-2-hydroxyquinic acid, a phenolic compound from needles of *Cedrus deodara*, on cellular functions of *Staphylococcus aureus*

**DOI:** 10.1039/c7ra13457f

**Published:** 2018-01-29

**Authors:** Yanping Wu, Jinrong Bai, Xiaoyan Liu, Lijin Liu, Kai Zhong, Yina Huang, Hong Gao

**Affiliations:** Healthy Food Evaluation Research Center, Sichuan University Chengdu 610065 China gao523@hotmail.com +86-28-8540-5236 +86-28-8550-3693; Department of Food Science and Technology, College of Light Industry, Textile and Food Engineering, Sichuan University Chengdu 610065 China dir0932@sina.com; Department of Public Health, West China Medical School, Sichuan University Chengdu 610041 China

## Abstract

A natural phenolic compound, 3-*p-trans*-coumaroyl-2-hydroxyquinic acid (CHQA) from needles of *Cedrus deodara*, has been reported to exhibit strong antibacterial activity. In this study, the molecular structural requirements of CHQA for the antibacterial activity and its effect on the cellular functions of *Staphylococcus aureus* were investigated. The structure–activity relationship analysis revealed that the *p*-coumaric acid moiety of CHQA was critical for the antibacterial activity, while the esterification between *p*-coumaric acid and 2-hydroxyquinic acid was unfavorable. Studies of the cellular metabolism demonstrated that CHQA induced a significant decrease in the intracellular ATP concentration but no proportional increase in the extracellular ATP. It was also found that CHQA slightly increased the respiratory activity and succinate dehydrogenase activity of *S. aureus*. Meanwhile, CHQA decreased the DNA synthesis of *S. aureus* and directly interacted with DNA through the groove binding mode.

## Abstract 2

Structure–activity relationship of 3-*p-trans*-coumaroyl-2-hydroxyquinic acid, a phenolic compound from needles of *Cedrus deodara*, against *Staphylococcus aureus* and its effect on the cellular functions.

## Graphical abstract

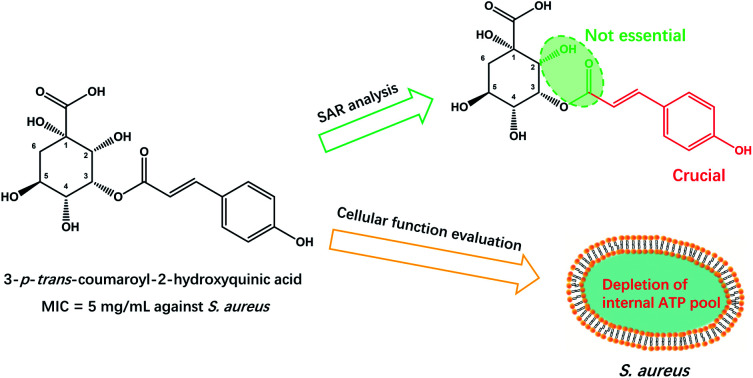

## 1. Introduction

Food spoilage and food poisoning provoked by foodborne pathogens are still an important and pressing global public health concern facing the food industry and consumers.^1,2^*Staphylococcus aureus* is one of the most significant foodborne pathogens and widely distributed in many food products, such as meat, milk, eggs, and bakery products.^3^*S. aureus* can cause a series of diseases, including skin infections, endocarditis, pneumonia, osteomyelitis, meningitis, septicemia, toxic shock syndrome, and staphylococcal food poisoning.^4,5^ The survival of *S. aureus* in foods puts consumers at high risk and imposes grave economic losses to producers. So, it is important to control and eliminate the growth of *S. aureus* in food to secure food quality and safety of consumers.

To date, many attempts have been made to prevent the microbial growth in food, and using food preservatives is one of the most important strategies. However, the extensive application of synthetic chemicals is currently controversial due to their potential harmful effects on human health, such as carcinogenesis, teratogenesis, respiratory hazard, and residual toxicity.^6,7^ Meanwhile, consumers are increasingly desiring natural and nutrient food with few perceived synthetic preservatives, which has inspired a great interest in development of safe and effective antimicrobial agents from natural sources as alternative preservatives.^8,9^ Phenolic compounds, common plant secondary metabolites, have gained much attention because of their antimicrobial properties against a broad range of foodborne pathogens.^2^

**Fig. 1:**
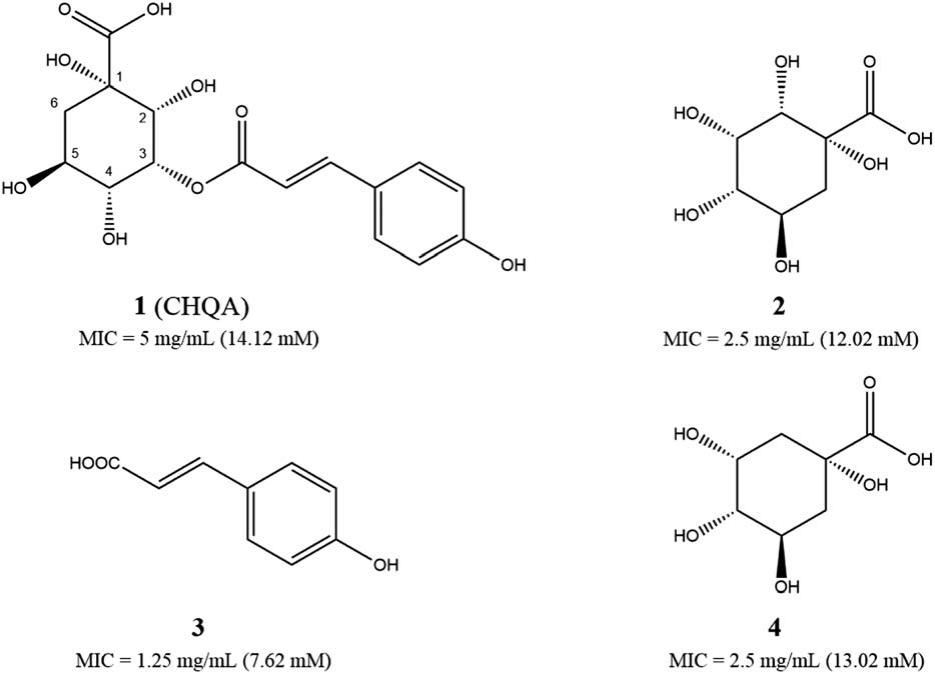
Chemical structures of 3-*p-trans*-coumaroyl-2-hydroxyquinic acid CHQA (1), 2-hydroxyquinic acid (2), *p*-coumaric acid (3) and quinic acid (4), and their minimum inhibitory concentration (MIC) values against *S. aureus* ATCC 6538.

We have recently reported a phenolic compound, 3-*p-trans*-coumaroyl-2-hydroxyquinic acid (CHQA, Fig. 1) from needles of *Cedrus deodara*, which showed strong antibacterial activity against both Gram-positive and Gram-negative bacteria.^10,11^ CHQA contains a 2-hydroxyquinic acid moiety together with an ordinary *p*-coumaric acid moiety, and it is unclear which moiety or chemical group is the critical active structure for the antibacterial activity. The preliminary mechanism study has demonstrated that CHQA performed its antibacterial activity by damaging the bacterial membrane along with significant hyperpolarization, loss of membrane integrity, severe morphological change, and interaction with membrane proteins and lipids.^10^ However, the effect of CHQA on the cellular metabolism of bacteria remains elusive.

Therefore, the aims of this study were to clarify the structural requirements of CHQA for the antibacterial activity, and to further investigate the effect of CHQA on the cellular functions of *S. aureus* by measuring the intracellular and extracellular ATP concentrations, respiratory activity, succinate dehydrogenase activity, DNA content, and interaction between CHQA and *S. aureus* DNA.

## 2. Materials and methods

### 2.1. Materials

CHQA (HPLC purity ≥ 98%) was purified from needles of *Cedrus deodara* according to our previous method.^11^ Quinic acid and *p*-coumaric acid (HPLC purity ≥ 98%) were purchased from Sigma Aldrich Co. (St. Louis, MO, USA). *Staphylococcus aureus* ATCC 6538 was obtained from the China Medical Culture Collection Center (Beijing, China). Nutrient broth and Mueller Hinton broth were purchased from Beijing Aoboxing Biotech Co. Ltd. (Beijing, China). All other chemicals used were of analytical grade.

### 2.2. Preparation of 2-hydroxyquinic acid

To a stirred solution of CHQA (30 mg, 0.085 mmol) in methanol (0.4 mL) was added dropwise 20% sodium ethoxide–ethanol solution (0.2 mL). The reaction mixture was kept at −20 °C over a period of 3 h and then at 4 °C overnight. The mixture was put through a IR-120 ion exchange column chromatography (2 × 6 cm, H&E Co. Ltd., Beijing, China) using pure water as eluent. The collected fraction was further purified by semi-preparative HPLC (mobile phase, water–methanol–formic acid (50 : 50 : 0.1); flow rate: 1 mL min^−1^; *t*_R_: 3.8 min; detection: 210 nm) with a Shimadzu LC-6AD system (Shimadzu, Co., Kyoto, Japan), using an Inertsil ODS column (4.6 × 250 mm, 5 μm, GL-Science Inc., Tokyo, Japan) to give 2-hydroxyquinic acid as a white powder: [*α*]^20^_D_ = −16° (*c* 1.0 in H_2_O); ESI-MS *m*/*z* 206.93 [M − H]^−^; ^1^H NMR *δ* (600 MHz, D_2_O) ppm: 4.08 (t, 1H, *J* = 3.3 Hz), 4.02 (d, 1H, *J* = 3.3 Hz), 3.92–3.96 (m, 1H), 3.51 (dd, 1H, *J* = 9.8, 3.3 Hz), 2.11 (dd, 1H, *J* = 13.7, 4.9 Hz), 1.78 (t, 1H, *J* = 13.7 Hz).

### 2.3. Determination of minimum inhibitory concentration

The MIC values of CHQA, 2-hydroxyquinic acid, *p*-coumaric acid and quinic acid against *S. aureus* were determined with broth microdilution method as previously reported.^10^ Briefly, *S. aureus* was cultured in nutrient broth overnight at 37 °C to obtain cells in the logarithmic phase, and diluted in Mueller Hinton broth to a density of 1 × 10^6^ CFU mL^−1^. Subsequently, 100 μL of the serial twofold dilutions of test compounds in Mueller Hinton and 100 μL of the bacterial suspension were added into a sterile 96-well microplate. The plate was then incubated at 37 °C for 24 h. The MIC value was reported as the lowest concentration of compound with no visible bacterial growth.

### 2.4. Measurement of intracellular and extracellular ATP concentrations

The intracellular and extracellular ATP concentrations of *S. aureus* were determined according to the method described previously with some modifications.^4^ Briefly, logarithmic phase *S. aureus* cells were harvested by centrifugation at 3000 rpm for 5 min, washed twice and resuspended in 0.85% sterile saline at a cell density of 1 × 10^9^ CFU mL^−1^. The bacterial suspensions were treated with different concentrations of CHQA at 37 °C for 30 min. Then, the samples were centrifuged at 5000 rpm for 5 min, the supernatants and cell pellets were stored on ice respectively to prevent ATP loss. The ATP concentration of the supernatants, which represents the extracellular concentration, was determined using an ATP assay kit (Life Technologies, Eugene, OR, USA) following manual instructions with a microplate reader (PE envision, Perkin Elmer Co., Waltham, MA, USA). To measure the intracellular ATP concentration, the cell pellets were washed, resuspended in 0.85% sterile saline, and treated by ultrasound on ice. After centrifugation at 10 000 rpm for 3 min, the ATP concentration of the resultant supernatants, which represents the intracellular concentration, was determined as described for the extracellular ATP concentration.

### 2.5. Flow cytometric analysis for respiratory activity

The effect of CHQA on the respiratory activity of *S. aureus* was assessed using flow cytometry coupled with 5-cyano-2,3-ditolyl tetrazolium chloride (CTC) stain (Sigma Aldrich Co., St. Louis, MO, USA), as previously reported.^12^ Briefly, logarithmic phase *S. aureus* cells were collected, washed and resuspended in PBS (0.01 M, pH 7.2) at a cell density of 1 × 10^9^ CFU mL^−1^. Aliquots of 1 mL of the bacterial suspensions were incubated with different concentrations of CHQA at 37 °C for 30 min. Then, samples were centrifuged at 5000 rpm for 5 min, washed and resuspended in 1 mL of PBS. The bacterial suspensions were further stained with 5 mM CTC for 30 min at 37 °C in the dark. After diluted ten times with ultrapure water, the cell suspensions were subjected to flow cytometric analysis on a BD FACSVerse flow cytometer (Becton Dickinson, San Jose, CA, USA). The fluorescence signal was detected by FL3 channel with a 620 nm bandpass filter. Data acquisition was set to 50 000 events for each sample. Suspensions of untreated and heat-killed for 30 min at 70 °C cells were served as the negative control and the positive control, respectively.

### 2.6. Measurement of succinate dehydrogenase activity

The succinate dehydrogenase (SDH) activity of *S. aureus* cells was measured as the previous reported method with minor exceptions.^13^ In brief, logarithmic phase *S. aureus* cells were collected, washed with 0.85% sterile saline and adjusted to a cell density of 1 × 10^9^ CFU mL^−1^. The bacterial suspensions were incubated with different concentrations of CHQA at 37 °C for 3 h. Subsequently, the SDH activity of *S. aureus* cells were assessed using the SDH Activity Colorimetric Assay Kit (GenMed Scientifics Inc., Shanghai, China) according to the manufacturer's protocols, using a microplate reader (Multiskan GO, Thermo Fisher Scientific Co., Waltham, MA, USA).

### 2.7. DAPI staining assay

The DNA content of *S. aureus* was determined by 4′,6-diamidino-2-phenylindole (DAPI, Sigma Aldrich Co., St. Louis, MO, USA) staining assay as the previous reported method with some modifications.^14^ Briefly, overnight culture of *S. aureus* was diluted with fresh nutrient broth to obtain a bacterial suspension of 1 × 10^9^ CFU mL^−1^. Aliquots of 1 mL of the bacterial suspensions were subjected to treatment with different concentrations of CHQA at 37 °C for 3 h. After treatment, cells were collected by centrifugation at 3000 rpm for 5 min, washed and resuspended in 1 mL of PBS. Then, cells were stained with 2.5 μg mL^−1^ of DAPI and incubated at 25 °C for 30 min. The stained bacteria were trapped on microscope slide and observed using an inverted fluorescence microscope (Nikon Ti-U, Nikon Corporation, Tokyo, Japan).

### 2.8. DNA binding assay

#### 2.8.1. Preparation of *S. aureus* genomic DNA

*S. aureus* genomic DNA was extracted as the previous reported method with minor modifications.^15^ The concentration and purity of the DNA were determined according to the OD_260 nm_ value, and the ratios OD_260 nm_/OD_280 nm_ and OD_260 nm_/OD_230 nm_ (1.8 ≤ OD_260 nm_/OD_280 nm_ ≤ 2.0, 2.0 ≤ OD_260 nm_/OD_230 nm_ ≤ 2.2), respectively, with a NanoDrop 1000 spectrophotometer (Thermo Fisher Scientific, Wilmington, DE, USA).

#### 2.8.2. Ultraviolet visible absorption study

Interaction of CHQA with the genomic DNA of *S. aureus* was investigated according to a UV-vis spectrophotometer method with some modifications.^16^ Absorption titrations of CHQA with *S. aureus* genomic DNA were performed using fixed concentration of CHQA (50 μg mL^−1^) in PBS with increasing amounts of DNA over a range of 0–16 μg mL^−1^. After incubation at 25 °C for 10 min, the UV-vis spectra of CHQA in the presence of different concentrations of DNA were measured, using a Shimadzu UV-2450 spectrophotometer (Tokyo, Japan).

#### 2.8.3. Fluorescence spectroscopic study

The competitive binding experiments were carried out with a slightly modified method.^16^ Briefly, different amounts of CHQA were added to the DNA–EB complex in PBS containing a fixed concentration of DNA (45 μg mL^−1^) and EB (1.25 μg mL^−1^). After incubation at 25 °C for 30 min, the fluorescence emission spectra were measured over a wavelength range of 550–750 nm with excitation at 530 nm using a fluorescence spectrophotometer (Cary Eclipse G9800A, Agilent technologies trading Co., Ltd., Shanghai, China).

### 2.9. Statistical analysis

All experiments were conducted in triplicate. Data were expressed as means ± SD. One-way analysis of variance and Duncan's multiple range tests were performed on SPSS 20.0 software (IBM Co., Armonk, NY, USA). Differences between means were considered statistically significant at *p* < 0.05.

## 3. Results and discussion

### 3.1. Structure–activity relationship of CHQA against *S. aureus*

The antibacterial activities of CHQA (1), 2-hydroxyquinic acid (2), *p*-coumaric acid (3) and quinic acid (4) against *S. aureus* were evaluated and described with minimum inhibitory concentration (MIC) values in Fig. 1. All of the test compounds showed inhibitory activity against *S. aureus* with MIC values over a range of 7.6–14.1 mM. The antibacterial activity of CHQA was comparable to that of 2-hydroxyquinic acid, while it was less active than that of *p*-coumaric acid. The results indicated that the *p*-coumaric acid moiety was critical for the antibacterial activity of CHQA, while the esterification between *p*-coumaric acid and 2-hydroxyquinic acid reduced the inhibitory activity. The findings were consistent with a previous study which reported that the antibacterial activity of caffeic acid against *S. aureus* was more potent than that of chlorogenic acid.^17^ Moreover, elimination of the hydroxyl group at position 2 of 2-hydroxyquinic acid did not affect the antibacterial activity by comparing the MIC values of 2-hydroxyquinic acid and quinic acid, which suggested that the hydroxyl group at position 2 of 2-hydroxyquinic acid was not essential for its inhibitory activity against *S. aureus*. Furthermore, it is fair to speculate that the hydroxyl group at position 2 of CHQA was also not essential for the antibacterial activity against *S. aureus*.

### 3.2. Effect of CHQA on the intracellular and extracellular ATP concentrations of *S. aureus*

**Fig. 2:**
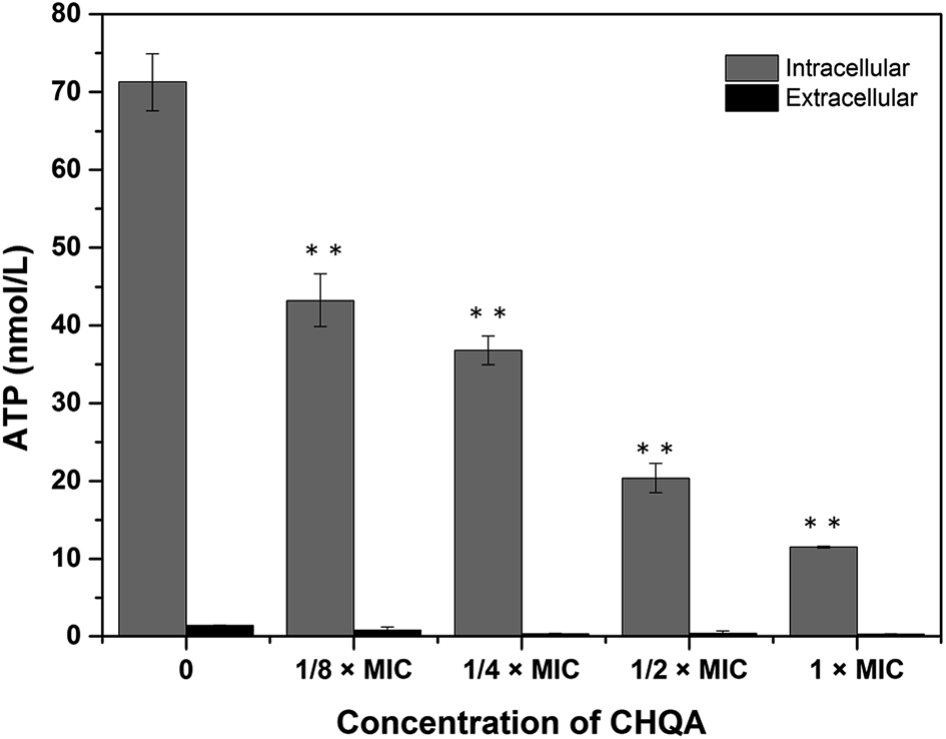
Effect of CHQA on the intracellular and extracellular ATP concentrations of *S. aureus* ATCC 6538. Bars represent the standard deviation (*n* = 3). **p < 0.01.

The effect of CHQA on the intracellular and extracellular ATP concentrations of *S. aureus* was presented in Fig. 2. The intracellular and extracellular ATP concentrations of untreated *S. aureus* cells were detected to be 71.27 ± 3.65 and 1.38 ± 0.07 nmol L^−1^, respectively. After treatment with 1/4 × MIC, 1/2 × MIC, and 1 × MIC of CHQA, the intracellular ATP concentrations of *S. aureus* were 36.80 ± 1.85, 20.40 ± 1.85, and 11.50 ± 0.10 nmol L^−1^, respectively, and the extracellular ATP concentrations were 0.98 ± 0.33, 1.13 ± 0.22, and 1.08 ± 0.24 nmol L^−1^, respectively. The results showed that *S. aureus* cells treated with CHQA from 1/4 × MIC to 1 × MIC showed a significant (*p* < 0.01) dose-dependent decrease in intracellular ATP concentration, while no significant (*p* > 0.05) difference was observed in the extracellular ATP concentration. There was no proportional increase in extracellular ATP concentration when the level of intracellular ATP decreased, which suggested that CHQA did not enhance the membrane permeability of *S. aureus* cells for the passage of ATP molecules. Thus, depletion of the internal ATP pool caused by CHQA might result from a decreased ATP synthesis and/or increased ATP hydrolysis.^18^ These results were in good agreement with previous work which demonstrated that chlorogenic acid decreased the intracellular ATP concentration of *S. aureus*, while no increase of extracellular ATP concentration was observed.^4^ In contrast, it was reported that *Escherichia coli* and *Salmonella typhi* showed a correlation between the intracellular and extracellular ATP concentrations after treatment with mustard essential oil.^19^ These differences were probably related to the various molecular mechanisms of action of different antibacterial agents and different membrane structures between Gram-positive and Gram-negative bacteria.

### 3.3. Effect of CHQA on the respiratory activity of *S. aureus*

**Fig. 3:**
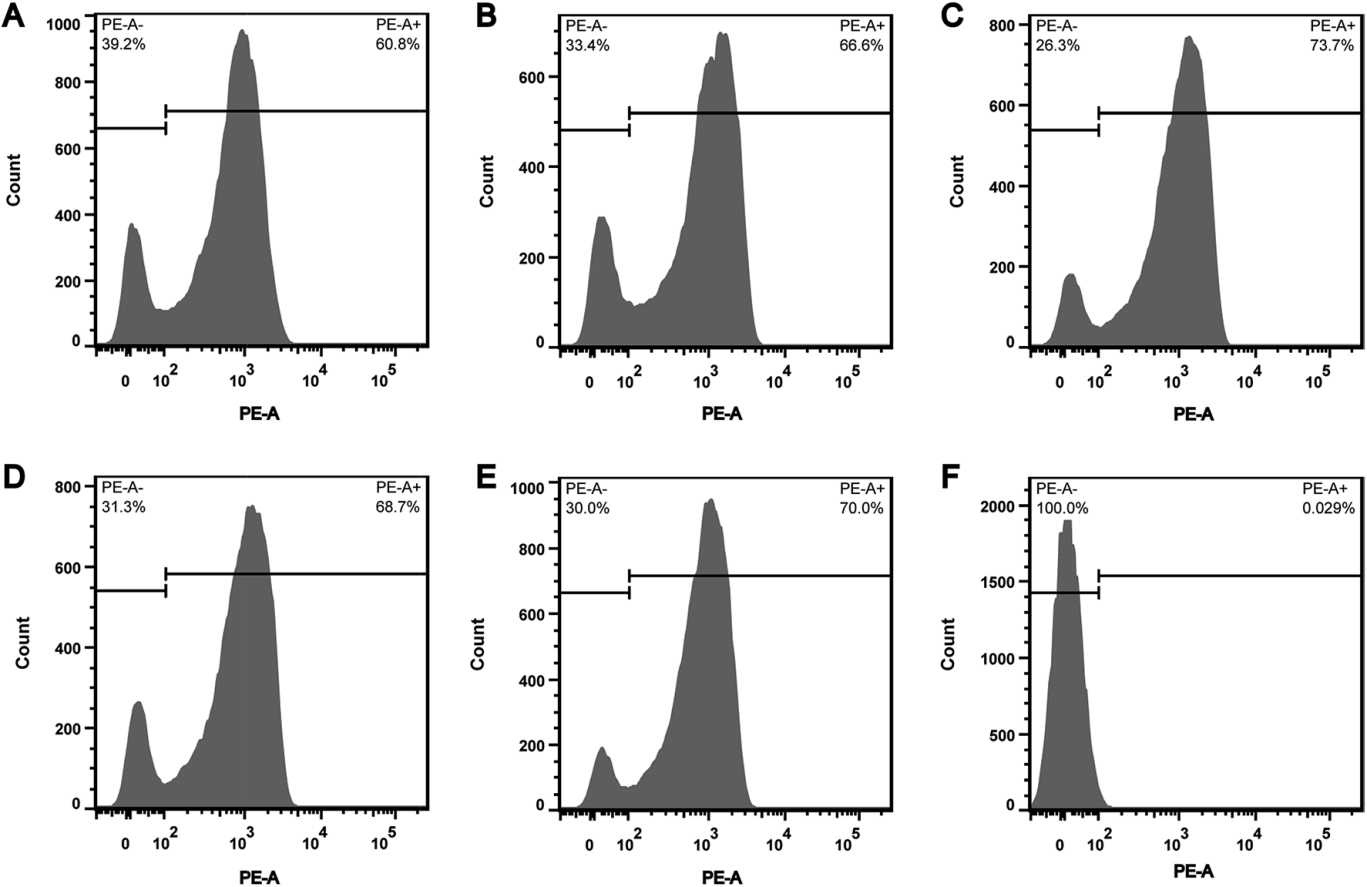
Effect of CHQA on the respiratory activity of *S. aureus* ATCC 6538. The cells within the PE-A^−^ region were taken as respiratory inactive bacteria, and those within the PE-A^+^ region were taken as actively respiring bacteria. Untreated bacteria (A); bacteria treated with CHQA at 1/4 × MIC (B), 1/2 × MIC (C), 1 × MIC (D) and 2 × MIC (E) for 30 min; and bacteria heated at 70 °C for 30 min (F).

In order to explore whether the depletion of internal ATP pool was caused by the reduction of ATP synthesis, the effect of CHQA on the respiratory activity of *S. aureus* was evaluated. CTC is a monotetrazolium redox dye which is practically colourless and nonfluorescent.^20^ Bacterial cells with respiratory activity or electron transport system activity are able to reduce CTC to an insoluble red fluorescent CTC–formazan product, which accumulates inside cells and can be quantified.^12,20^ As shown in the histogram (Fig. 3), two regions of CTC–formazan relative fluorescence were defined: PE-A^−^ (respiratory inactive bacteria region) and PE-A^+^ (actively respiring bacteria region). In the untreated *S. aureus* (Fig. 3A), the percentage of cells that were able to reduce CTC was 60.8%. This was in accordance with a previous study which detected that the negative control suspension of *Pseudomonas aeruginosa* contained 66.9% of CTC-reducing bacteria.^21^ The detection of relatively low proportion of CTC-reducing cells in negative control probably explained by the fact that bacteria with a low respiratory activity may not be detected as CTC-positive.^22^ Indeed, other authors have also suggested that CTC has a toxic effect on bacteria, thereby resulting in underestimation of the proportion of actively respiring cells.^23,24^ Compared with the negative control, a minor increase in the capacity of *S. aureus* cells to reduce CTC was observed after incubation with CHQA at concentrations from 1/4 to 2 × MIC, whereas the heat treatment caused nearly all cells to lose respiratory activity (Fig. 3). One possible reason is that the minor increase of respiratory activity was associated with the attempt of *S. aureus* to compensate for the depletion of intracellular ATP under the chemical stress environment induced by CHQA, and a 30 min of contact with CHQA could not directly damage the metabolic system of bacteria. The results suggested that CHQA could not decrease the ATP synthesis of *S. aureus*. Therefore, it was implied that the significant reduction of intracellular ATP of *S. aureus* might occur as a consequence of increased ATP hydrolysis rather than decreased ATP synthesis.

### 3.4. Effect of CHQA on the succinate dehydrogenase activity of *S. aureus*

**Fig. 4:**
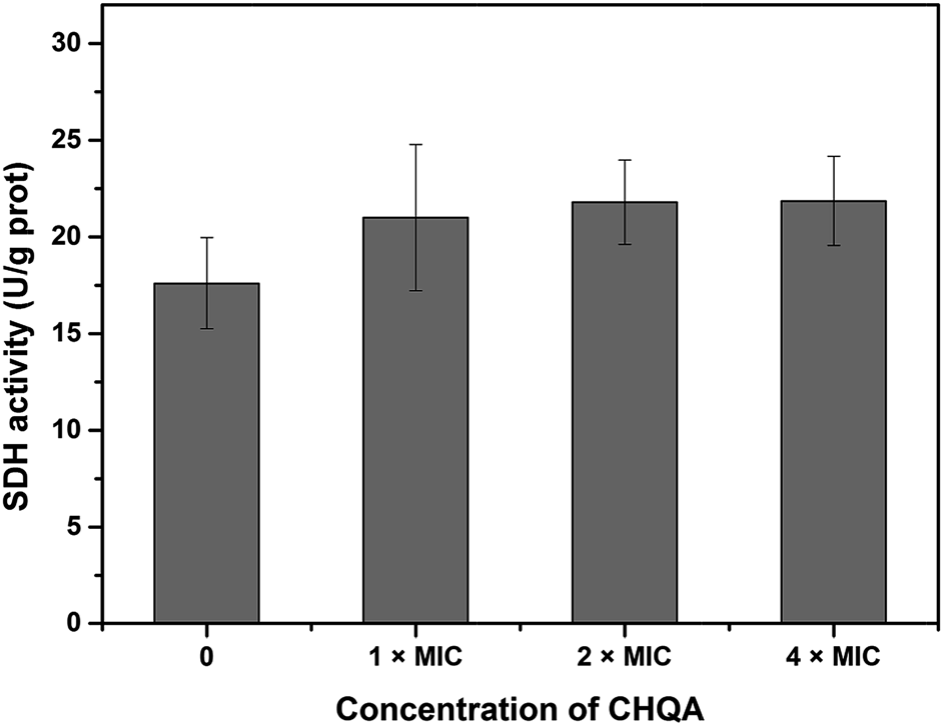
Effect of CHQA on the succinate dehydrogenase activity of *S. aureus* ATCC 6538. Bars represent the standard deviation (*n* = 3).

Succinate dehydrogenase, a key catalyzing enzyme in tricarboxylic acid cycle, is greatly important for the bioenergy synthesis of bacteria and its activity reflects the energy metabolic status of bacterial cell.^13^ To further validate the effect of CHQA on the metabolic activity of *S. aureus*, the SDH activity was measured and presented in Fig. 4. The SDH activity of untreated *S. aureus* cells was 17.6 ± 2.3 U g^−1^ protein. On the other hand, the SDH activity of *S. aureus* cells treated with CHQA at 1 × MIC, 2 × MIC and 4 × MIC was 21.0 ± 3.8, 21.8 ± 2.2, 21.9 ± 2.3 U g^−1^ protein, respectively. The results showed that CHQA slightly increased the SDH activity of *S. aureus*, while no significant difference (*p* > 0.05) was observed even at high concentrations. These findings were consistent with the flow cytometric analysis which indicated that the treatment of CHQA caused a minor increase in the respiratory activity of *S. aureus*. Combined with these results, it was suggested that CHQA nearly did not affect the ATP synthesis pathway of *S. aureus*. Moreover, the significant reduction of intracellular ATP concentration induced by CHQA might be due to the increased rate of ATP hydrolysis. However, the direct target molecules of CHQA against *S. aureus* that result in the reduction of intracellular ATP need to be further investigated.

### 3.5. Effect of CHQA on the DNA synthesis of *S. aureus*

**Fig. 5:**
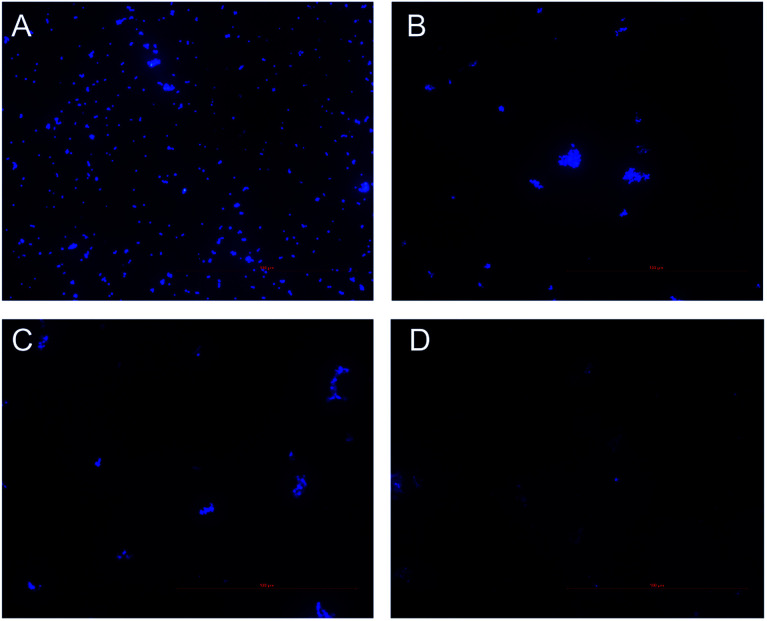
Effect of CHQA on the DNA synthesis of *S. aureus* ATCC 6538. Untreated bacteria (A); bacteria treated with CHQA at 1/4 × MIC (B), 1/2 × MIC (C), and 1 × MIC (D) at 37 °C for 3 h.

Inhibition of nucleic acid synthesis is another important antibacterial mechanism of natural compounds. So, the effect of CHQA on the DNA synthesis of *S. aureus* was evaluated to further elucidate its mode of action. DAPI is a specific fluorescence dye for nucleic acid, and DNA–DAPI complex emits blue fluorescence when excited with appropriate UV-light.^25,26^ The fluorescence intensity enhanced with the increase in content of DNA. As illustrated in Fig. 5, the microscopy images showed that the fluorescence intensity of the CHQA treated group was obviously weaker than that of the control group, and the fluorescence intensity decreased along with the increasing concentration of CHQA from 1/4 × MIC to 1 × MIC. It was indicated that CHQA could markedly restrain the DNA synthesis of *S. aureus*, and DNA would be the target molecule inside the bacterial cell. In a previous study, similar findings have been reported in *Actinobacillus pleuropneumoniae* cells treated with berberine which showed strong inhibitory effect against DNA synthesis.^14^ DNA, one of the most basic life molecules, has a decisive effect on gene expression, heredity and variation. The potent inhibitory effect of CHQA on the DNA synthesis would give rise to cellular dysfunction of *S. aureus*, thus leading to growth inhibition or even death.

### 3.6. DNA binding studies

#### 3.6.1. UV-vis absorption spectral studies

**Fig. 6:**
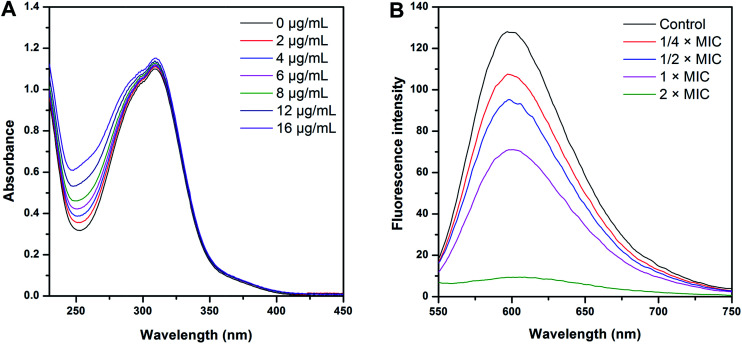
Spectroscopic analysis of the interaction of CHQA with the genomic DNA of *S. aureus* ATCC 6538. (A) Ultraviolet spectra of CHQA in the absence and presence of increasing amounts of DNA; (B) fluorescence spectra of the DNA–EB system in the absence and presence of increasing amounts of CHQA.

Generally, for small molecular compounds which bind to DNA noncovalently, intercalation and groove binding are the two most likely binding modes.^27^ Intercalation, in which a planar ligand moiety is inserted between adjacent base pairs, is stabilized electronically by π–π stacking and dipole–dipole interactions, and results in a substantial change in DNA structure.^27,28^ In contrast, groove binding is stabilized by electrostatic, hydrophobic and hydrogen-bonding interactions and typically causes only subtle changes in DNA structure.^27,28^ UV-vis spectroscopic titration is an effective and convenient method to examine the mode of interaction. Absorption titration was performed by maintaining a constant concentration of CHQA while increasing the DNA concentration. As shown in Fig. 6A, addition of increasing amounts of DNA caused a tiny increase in absorbance around 310 nm, and no hypochromic effect was observed. Moreover, the maximum absorption wavelength of CHQA at 310 nm almost did not change. Similar observation has also been reported previously by Qiao *et al.* for the interaction of anthraquinones with fish sperm DNA.^29^ These results implied that the existence interaction between CHQA and *S. aureus* DNA, and the interactive mode were not the classical intercalation binding.

#### 3.6.2. Fluorescent spectral studies

The binding characteristics of CHQA with *S. aureus* DNA in the presence of ethidium bromide (EB) were further investigated using fluorescence spectroscopy. EB, a cationic conjugated planar molecule, is well known as a DNA intercalator with intercalation of the planar phenanthridinium ring between the adjacent base pairs of double-stranded DNA.^30^ The fluorescence intensity of EB in aqueous environments is usually weak, whereas it is greatly enhanced when EB intercalates into DNA.^27^ Thus, EB has been widely used as a probe for the spectroscopic study of interaction between DNA and small molecular compounds.^31^ The fluorescence spectra of DNA–EB complex in the absence and presence of CHQA were shown in Fig. 6B. Obviously, the fluorescence of DNA–EB complex was quenched dramatically by CHQA, and the fluorescence intensity decreased with increasing concentration of CHQA. The UV-vis spectroscopic assay suggested that the interactive mode between CHQA and *S. aureus* DNA was not classical intercalation binding, thus there almost impossibly existed competition between CHQA and EB. Consequently, the quenching effect of CHQA was probably attributed to the binding of CHQA to the DNA–EB complex and forming a new shape of non-fluorescence complex DNA–EB–CHQA, resulting in the decrease of fluorescence intensity. Indeed, similar fluorescence quenching effects on the DNA–EB system has been observed for some groove binding compounds, such as netropsin, distamycin A, chrysophanol, and physcion.^29,32^ Therefore, it is reasonable to conclude that CHQA may interact with *S. aureus* DNA through the groove binding mode. Previously, we have demonstrated that CHQA possessed a membrane-disruptive mechanism against *S. aureus*.^10^ Combined with these results, it was proposed that CHQA achieved the antibacterial activity through multi-target mechanism.

## 4. Conclusions

In this study, the antibacterial mechanism of CHQA against *S. aureus* was elucidated in terms of the molecular chemical requirements of CHQA and its effect on the cellular functions by exploring ATP concentration, respiratory activity, SDH activity, DNA synthesis, and interaction between CHQA and *S. aureus* DNA. The structure–activity relationship analysis suggested that the *p*-coumaric acid moiety of CHQA was critical for the inhibitory activity against *S. aureus*, while the esterification between *p*-coumaric acid and 2-hydroxyquinic acid was unfavourable. The antibacterial activity of CHQA might be partially achieved by increasing the ATP hydrolysis of *S. aureus* cell, as evidenced by that CHQA induced a significant decrease in intracellular ATP concentration, no proportional increase in extracellular ATP concentration, and minor increase in respiratory activity and SDH activity. Moreover, CHQA dramatically restrained the DNA synthesis of *S. aureus* and interacted with DNA through the groove binding mode, resulting in cellular dysfunction and even bacteria death. However, further studies on the toxicity of CHQA to human cell lines and influence on the food sensory properties are necessary for the future application in food products.

## Conflicts of interest

There are no conflicts to declare.

## Supplementary Material
